# Circulating GDF15 May Estimate Vasculitis Activity and Predict Poor Outcomes During the Disease Course of ANCA-Associated Vasculitis

**DOI:** 10.3390/jcm14061876

**Published:** 2025-03-11

**Authors:** Taejun Yoon, Jang Woo Ha, Yong-Beom Park, Sang-Won Lee

**Affiliations:** 1 Division of Rheumatology, Department of Internal Medicine, Yonsei University College of Medicine, Seoul 03722, Republic of Korea; 2 Division of Rheumatology, Department of Internal Medicine, Yongin Severance Hospital, Yonsei University College of Medicine, Yongin 16995, Republic of Korea; 3 Institute for Immunology and Immunological Diseases, Yonsei University College of Medicine, Seoul 03722, Republic of Korea

**Keywords:** GDF15, activity, mortality, end-stage kidney disease, antineutrophil cytoplasmic antibody

## Abstract

**Objective:** This study investigated whether circulating growth differentiation factor 15 (GDF15) at diagnosis could estimate the Birmingham Vasculitis Activity Score (BVAS) and potentially predict all-cause mortality and end-stage kidney disease (ESKD) during follow-up in patients with antineutrophil cytoplasmic antibody (ANCA)-associated vasculitis (AAV). **Methods:** This study included 79 patients selected from a cohort of Korean patients with AAV. Circulating GDF15 was measured from patients’ sera collected at diagnosis and stored at −80 °C. Clinical data at diagnosis and during follow-up were reviewed. **Results:** The median age was 64.0 years (40.5% men, and 59.5% women). Median circulating GDF15 was measured as 995.0 pg/mL. Of the 79 patients, 6 (7.6%) died and 20 (25.3%) progressed to ESKD during the disease course. Circulating GDF15 levels were significantly correlated with BVAS (r = 0.340) at diagnosis. Patients with circulating GDF15 ≥ 3350.5 pg/mL exhibited a significantly higher risk of the highest tertile of BVAS than those without (relative risk [RR], 11.229). Similarly, patients with circulating GDF15 ≥ 2239.5 pg/mL and ≥2208.5 pg/mL showed higher risks of all-cause mortality (RR, 7.733) and progression to ESKD (RR 7.125) than those without. Patients with circulating GDF15 ≥ 2239.5 pg/mL and ≥2208.5 pg/mL also showed significantly lower patient and ESKD-free survival rates than those without. **Conclusions:** Circulating GDF15 at diagnosis is useful in estimating BVAS and potentially predicts all-cause mortality and ESKD progression in patients with AAV.

## 1. Introduction

Growth differentiation factor 15 (GDF15), first known as macrophage inhibitory cytokine 1, is part of the family of transforming growth factor-β [1]. During normal conditions, GDF15 is commonly expressed at low concentrations in almost all major organs.; however, in abnormal conditions or stressful situations, it may be upregulated predominantly in the liver, kidneys, heart, and lungs (and particularly in malignancies) and acts as a regulatory cytokine [2]. To date, GDF15 has been known to regulate inflammatory processes, apoptosis, angiogenesis, and cell growth and repair and was reported to play key roles in regulating body energy-related biological reactions, such as obesity and metabolic diseases [2,3,4]. Additionally, it has recently been unveiled that GDF15 may play important roles in the ageing process [5,6]. In contrast, circulating GDF15 has been introduced as a prognostic protein, and elevated concentrations of circulating GDF15 have been observed in atherosclerotic, ischaemic, and cardiac fibrotic conditions [7]; higher circulating GDF15 levels were found in patients with fibrotic hypersensitivity pneumonitis than in those with non-fibrotic hypersensitivity pneumonitis [8]. Serum GDF15 could be a predictor of 3-year poor outcomes of renal diseases [9]. Therefore, circulating GDF15 is currently accepted as a substance that is induced by chronic inflammation and is able to suppress/modulate the immune system involved in the inflammatory response; it is considered a factor that potentially predicts the prognosis of chronic inflammatory diseases through this mechanism.

Antineutrophil cytoplasmic antibody (ANCA)-associated vasculitis (AAV) belongs to a group of small vessel vasculitides and is characterised by fibrinoid-necrotising inflammation but with few or without immune deposits histologically [10]. Additionally, AAV is categorised into three subtypes based on the clinical, laboratory, radiological, and histological features: microscopic polyangiitis (MPA), granulomatosis with polyangiitis (GPA), and eosinophilic granulomatosis with polyangiitis (EGPA) [11,12,13,14]. The Birmingham Vasculitis Activity Score (BVAS) was used for assessing the cross-sectional AAV activity, and the Five-Factor Score (FFS) was applied for anticipating the future prognosis during the disease course. They include items for systemic clinical manifestations in various organs that are associated with an increase in circulating GDF15 when damage occurs [15,16,17]. Therefore, it could be theoretically speculated that circulating GDF15 might have clinical implications not only at diagnosis but also during the disease course of AAV. However, until now, no studies have investigated the clinical significance and implications of circulating GDF15 levels in patients with AAV. Hence, in this study, among immunosuppressive drug-free patients with AAV, we investigated whether circulating GDF15 levels measured at the time of AAV diagnosis can estimate the cross-sectional AAV activity represented by BVAS and have the potential to predict poor outcomes during AAV follow-up.

## 2. Materials and Methods

### 2.1. Patients

We randomly selected 79 immunosuppressive drug-free patients who were diagnosed with AAV and enrolled in a prospective and observational cohort of Korean patients with AAV at the Severance Hospital (the SHAVE cohort) between November 2016 and June 2023. In this study, ‘immunosuppressive drug-free patients with AAV’ were defined as patients who had never taken immunosuppressive drugs for AAV treatment within the 4 weeks before AAV diagnosis. All the patients fulfilled the 2007 European Medicine Agency algorithm for AAV, the 2012 revised International Chapel Hill Consensus Conference Nomenclature of Vasculitides, and the 2022 American College of Rheumatology/European Alliance of Associations for Rheumatology (2022 ACR/EULAR) classification criteria for AAV [10,11,12,13,14]. Medical records were sufficiently well documented for collecting clinical data from the time of AAV diagnosis to either a poor outcome occurrence or the last visit, and the follow-up period was at least 6 months or longer. No patients with serious medical conditions—such as malignancies or infectious diseases requiring hospitalization, which could mimic AAV manifestations and confound its diagnosis—were included [12,13,14,18,19]. Additionally, no patients with co-existing diseases or drug history affecting ANCA results were included [20,21].

This study was performed in accordance with the Declaration of Helsinki and approved by the Institutional Review Board (IRB) of the Severance Hospital, Seoul, Republic of Korea (IRB number 2024-0725-001). Written informed consent was obtained from all the patients at the time of blood sampling. Patients or the public were not involved in the design, conduct, reporting, or dissemination plans of our research.

### 2.2. Blood Sampling and Consent Form

On the same day as AAV diagnosis, patients handed in the informed consent forms for the provision of clinical data and blood samples. After obtaining informed consent, clinical data were recorded, and whole blood was collected. Sera were immediately isolated from whole blood and stored in the deep freezer at −80 °C.

### 2.3. Clinical Data at Diagnosis

In terms of demographic data, age, sex, smoking history, and body mass index were collected. In terms of AAV-specific variables, AAV subtype, ANCA positivity, AAV-specific indices including BVAS and FFS, and BVAS items were reviewed. Comorbidities such as type 2 diabetes mellitus, hypertension, and dyslipidaemia were investigated, and laboratory results, including erythrocyte sedimentation rate (ESR) and C-reactive protein (CRP) levels as acute-phase reactants, were also obtained.

### 2.4. Measurement of Circulating GDF15 at Diagnosis

The levels of circulating GDF15 were assessed using enzyme-linked immunosorbent assay kits (R&D Systems, Minneapolis, MN, USA) from sera.

### 2.5. Clinical Data During AAV Disease Course

Among the poor outcomes of AAV, death of any cause and end-stage kidney disease (ESKD) progression after AAV diagnosis were accepted as poor prognoses of AAV during follow-up. Follow-up duration based on each poor outcome was defined as the period from AAV diagnosis to its occurrence in patients with a corresponding poor outcome, whereas the duration from diagnosis to the last visit was defined for those without AAV. Medications, including glucocorticoids and immunosuppressive drugs, administered after the time of AAV diagnosis and during the disease course were also assessed.

### 2.6. ANCA Types

Myeloperoxidase (MPO)-ANCA and proteinase 3 (PR3)-ANCA were evaluated using immunoassays, and perinuclear (P)-ANCA and cytoplasmic (C)-ANCA were detected using indirect immunofluorescence assays. Based on the 2022 ACR/EULAR classification criteria for AAV [12,13,14], both MPO-/PR3-ANCAs and P-/C-ANCAs were accepted as ANCA test results. Therefore, when displaying ANCA test results, the variables were expressed as MPO-ANCA (or P-ANCA) and PR3-ANCA (or C-ANCA) in this study.

### 2.7. Statistical Analyses

All statistical analyses were performed using IBM SPSS Statistics for Windows version 26 (IBM Corp., Armonk, NY, USA). Continuous and categorical variables are expressed as medians (25–75 percentiles) and numbers (percentages). The correlation coefficient (r) between the two variables was obtained using Pearson correlation analysis. A significant area under the curve (AUC) was confirmed using receiver operator characteristic (ROC) curve analysis. The optimal cut-off was extrapolated by performing an ROC curve analysis and selected as that with the maximum sum of sensitivity and specificity. The relative risk (RR) of the cut-off for all-cause mortality was analysed using contingency tables and the chi-square test. The cumulative survival rates between the two groups were compared using the Kaplan–Meier survival analysis with the log-rank test. The multivariable Cox hazard model using variables with *p* < 0.1 in the univariable Cox hazard model was conducted to appropriately obtain the hazard ratios (HRs) during the considerable follow-up duration. Statistical significance was set at *p* < 0.05.

## 3. Results

### 3.1. Characteristics at AAV Diagnosis

The median age of the 79 patients was 64.0 years (40.5% men and 59.5% women). Among the 79 patients with AAV, 39 were diagnosed with MPA, 24 with GPA, and 16 with EGPA. MPO-ANCA (or P-ANCA) and PR3-ANCA (or C-ANCA) were positive in 43 (57.0%) and 12 (15.2%) patients, respectively. Circulating GDF15 levels were significantly correlated with MPO-ANCA titres (r = 0.567, *p* < 0.001) but not with PR3-ANCA titres (r = −0.148, *p* = 0.193). The median BVAS and FFS were 5.0 and 0, respectively, and the bottom of the highest tertile of BVAS was 12. The most frequent clinical manifestation based on BVAS items was pulmonary (62.0%) manifestation, followed by otorhinolaryngologic (51.9%) and renal (48.1%) manifestations. The median ESR and CRP were measured as 21.0 mm/h and 3.6 mg/L, respectively. The median circulating level of GDF15 was determined to be 995.0 pg/mL (Table 1).

### 3.2. Characteristics During the Disease Course

Among the patients in this study, 6 (7.6%) died after a follow-up duration of 26.7 months, whereas 20 (25.3%) experienced progression to ESKD for a follow-up duration of 25.8 months. A total of 78 patients received glucocorticoids, and the most commonly administered immunosuppressive drug was cyclophosphamide (65.8%), followed by azathioprine (60.8%) during the disease course of AAV (Table 1).

### 3.3. Correlation of Circulating GDF15 with AAV Activity-Related Variables at Diagnosis

**Figure 1:**
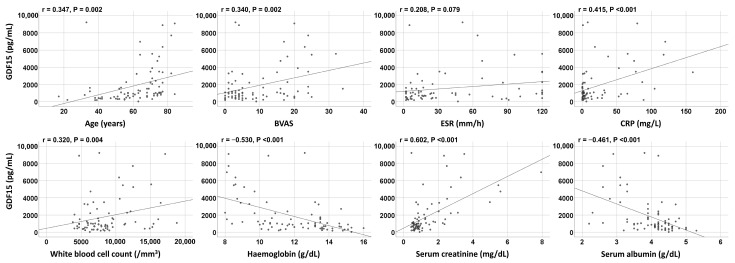
Correlation analyses. Circulating GDF15 was significantly correlated with BVAS and other variables at diagnosis. BVAS: Birmingham Vasculitis Activity Score; CRP: C-reactive protein; ESR: erythrocyte sedimentation rate.

Circulating GDF15 was significantly correlated with BVAS (r = 0.340). Additionally, age (r = 0.347), CRP levels (r = 0.415), white blood cell count (r = 0.320), and serum creatinine level (r = 0.602) exhibited significant and positive correlations with circulating levels of GDF15. Erythrocyte sedimentation rate tended to correlate with circulating GDF15, but it was not significant. Additionally, circulating GDF15 was inversely correlated with haemoglobin (r = −0.530) and serum albumin (r = −0.461) (Figure 1).

### 3.4. Optimal Cut-Off of Circulating GDF15 and Its Relative Risk to the Highest Tertile of BVAS at Diagnosis

**Figure 2:**
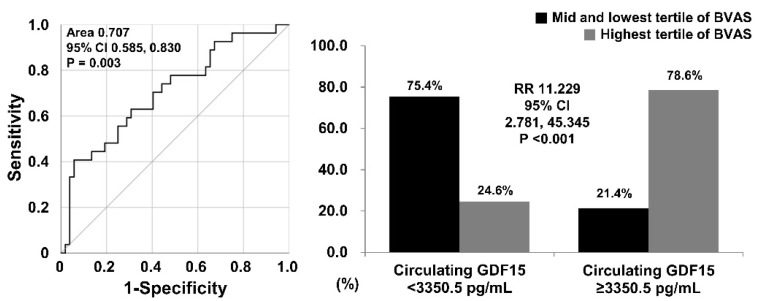
Cut-off and relative risk of circulating GDF15 of the highest tertile of BVAS. When the cut-off of circulating GDF15 for the highest tertile of BVAS was set at 3350.5 pg/mL, patients with circulating GDF15 ≥ 3350.5 pg/mL exhibited a significantly higher risk for the highest tertile of BVAS than those without (RR 11.229). BVAS: Birmingham Vasculitis Activity Score; RR: relative risk.

According to ROC curve analysis, when the cut-off of circulating GDF15 for the highest tertile of BVAS was set at 3350.5 pg/mL, the sensitivity and specificity were 40.7% and 94.2%, respectively (AUC 0.707, 95% confidence interval [CI] 0.585, 0.830). When patients were divided into two groups according to circulating GDF15 ≥ 3350.5 pg/mL, the highest tertile of BVAS was identified more frequently in patients with circulating GDF15 ≥ 3350.5 pg/mL than those with circulating GDF15 < 3350.5 pg/mL (78.6% vs. 24.6%, *p* < 0.001). Additionally, patients with circulating GDF15 ≥ 3350.5 pg/mL exhibited a significantly higher risk of the highest tertile of BVAS than those with circulating GDF15 < 3350.5 pg/mL (RR, 11.229; 95% CI: 2.781, 45.345) (Figure 2).

### 3.5. Determination of a Cut-Off of Circulating GDF15 at Diagnosis and Its Relative Risk for All-Cause Mortality During the Disease Course 

**Figure 3:**
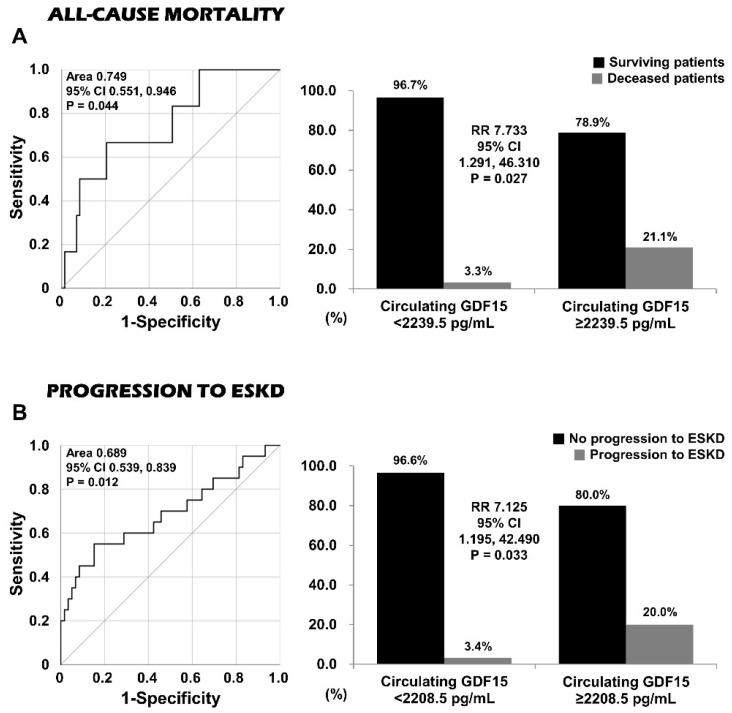
Cut-offs and relative risks of circulating GDF15 for all-cause mortality and progression to ESKD. Patients with circulating GDF15 ≥ 2239.5 pg/mL and those with circulating GDF15 ≥ 2208.5 pg/mL exhibited higher risks of all-cause mortality (RR 7.733) and progression to ESKD (RR 7.125) than those without. ESKD: end-stage kidney disease; RR: relative risk.

When the cut-off of circulating GDF15 at diagnosis for all-cause mortality during the disease course was determined to be 2239.5 pg/mL, the sensitivity and specificity were significant (66.7% and 79.5%; AUC, 0.749; 95% CI: 0.551, 0.946). Patients with circulating GDF15 ≥ 2239.5 pg/mL exhibited a higher frequency of all-cause mortality (21.1% vs. 3.3%, *p* = 0.027), and a significantly higher risk of all-cause mortality (RR, 7.733; 95% CI: 1.291, 46.310) compared with those with circulating GDF15 <2239.5 pg/mL (Figure 3A).

### 3.6. Determination of a Cut-Off of Circulating GDF15 at Diagnosis and Its Relative Risk for Progression to ESKD During the Disease Course

When the cut-off of circulating GDF15 at diagnosis for progression to ESKD during the disease course was determined to be 2208.5 pg/mL, the sensitivity and specificity were calculated as 55.0% and 84.7%, respectively (AUC, 0.689; 95% CI: 0.539, 0.839). Patients with circulating GDF15 ≥ 2208.5 pg/mL exhibited a higher frequency of progression to ESKD (20.0% vs. 3.4%, *p* = 0.033) and significantly higher risk of progression to ESKD (RR, 7.125; 95% CI: 1.195, 42.490) than those with circulating GDF15 <2208.5 pg/mL (Figure 3B).

### 3.7. Comparison of Cumulative Patients and ESKD-Free Survival Rates According to Each Cut-Off

**Figure 4:**
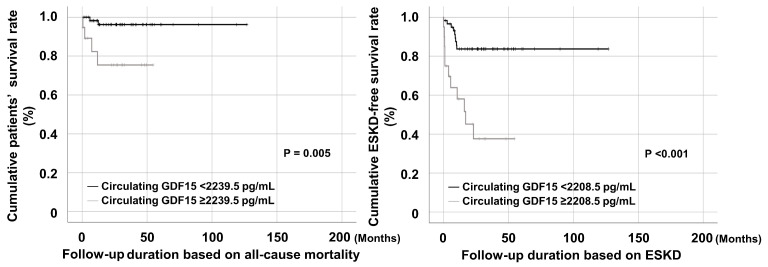
Cumulative survival rates. Patients with circulating GDF15 ≥ 2239.5 pg/mL and those with circulating GDF15 ≥ 2208.5 pg/mL also showed significantly lower patient and ESKD-free survival rates than those without. ESKD: end-stage kidney disease.

Patients with circulating GDF15 ≥ 2239.5 pg/mL exhibited a significantly lower patient survival rate than those with circulating GDF15 <2239.5 pg/mL (*p* = 0.005). Additionally, patients with circulating GDF15 ≥ 2208.5 pg/mL showed a significant reduction in the rate of ESKD-free survivals compared with those with circulating GDF15 <2208.5 pg/mL (*p* < 0.001) (Figure 4).

### 3.8. Cox Proportional Hazards Model Analyses of Variables at Diagnosis for All-Cause Mortality or Progression to ESKD During the Disease Course

The univariable Cox analysis for all-cause mortality revealed the significant associations of the variables of age, BVAS, CRP, white blood cell count, haemoglobin, total serum protein, serum albumin, and both circulating GDF15 and GDF15 of ≥2239.5 pg/mL at diagnosis with all-cause mortality during the disease course. Meanwhile, none of the variables with statistical significance upon univariable Cox analysis were independently associated with all-cause mortality during the disease course in multivariable Cox analysis. Conversely, upon univariable Cox analysis for progression to ESKD, female sex, ESR, CRP, haemoglobin, blood urea nitrogen, serum creatinine, serum albumin, and both circulating GDF15 and GDF15 of ≥2208.5 pg/mL at diagnosis exhibited an association with progression to ESKD during the disease course. Upon multivariable Cox analysis, both the ESR and serum creatinine levels at diagnosis were independently associated with ESKD progression during the disease course. In particular, circulating GDF15 of ≥2208.5 pg/mL, but not circulating GDF15, showed a tendency toward independent association with progression to ESKD during the disease course (HR, 3.979; *p* = 0.065); however, there was no statistical significance (Table 2 and Table S1).

## 4. Discussion

Given that circulating GDF15 has been considered a prognostic protein in several chronic diseases, we included 79 immunosuppressive drug-free patients with AAV in this study and investigated whether circulating GDF15 at diagnosis could estimate the cross-sectional BVAS and potentially foresee poor outcomes during the disease course of AAV. We obtained several findings. First, circulating GDF15 could estimate BVAS along with serum creatinine and acute-phase reactants, such as CRP levels and serum albumin at diagnosis. Second, a cut-off of circulating GDF15 for the highest tertile of BVAS was determined, which provided clinical utility in estimating the high activity of AAV at diagnosis. Third, the cut-offs of circulating GDF15 at diagnosis for all-cause mortality and progression to ESKD were set; they could predict the occurrence of all-cause mortality and progression to ESKD, respectively. Fourth, when above the cut-offs of circulating GDF15 were set at diagnosis for all-cause mortality and progression to ESKD, the survival benefit was significantly reduced compared to the opposites. However, their independent ability to predict poor outcomes was not demonstrated in the Cox analyses, despite the tendency toward an independent association between circulating GDF15 at diagnosis and progression to ESKD during the disease course. This study demonstrated that among patients with AAV, circulating GDF15 at diagnosis has clinical implications in estimating BVAS and the potential to predict death and ESKD occurrence in the disease course of AAV after diagnosis.

We were curious about the mechanism by which circulating GDF15 measured at diagnosis could reflect not only the extent of the inflammatory burden but also the BVAS at that time. To clarify this hypothesis, it is important to determine whether circulating GDF15 is a mediator that induces or reduces inflammation when inflammation occurs. First, in terms of the relationship between circulating GDF15 and inflammation, among the conflicting and controversial hypotheses, the most credible is that inflammation might induce GDF15, and in turn, GDF15 might act as an anti-inflammatory player in the setting of inflammation [22,23]. Various study results that could support and demonstrate this hypothesis have been reported. (i) In the intracellular signalling process, inflammation and stress stimuli may increase the gene expression of GDF15 through the pathways of various transcription factors, such as the nuclear factor kappa–light-chain-enhancer of activated B cells (NF-κB) and cAMP-responsive element binding protein 1 [23,24]; (ii) the produced GDF15 may be subsequently released out of the cell and finally become circulating GDF15 [23,25]; and (iii) circulating GDF15 can play an anti-inflammatory role by either indirectly turning on several additional intracellular signalling pathways—such as Smad, protein kinase B, and extracellular-regulated kinase—or directly inhibiting the NF-κB- c-Jun N-terminal kinases-caspase pathway [26].

Next, in terms of the link between circulating GDF15 and the organ-specific activity of AAV represented by BVAS, attention should be paid to the link between the concentration of circulating GDF15 and extent or severity of organ damage. Therefore, several previous studies have provided concrete evidence that GDF15 might play an antagonistic role against organ-specific inflammation; furthermore, it could alleviate inflammation in each organ included [25,26,27,28]. Additionally, circulating GDF15 at diagnosis is reportedly associated with poor outcomes or irreversible damage to major organs during the follow-up period [29,30]. Taken together with these findings, there is a sufficient theoretical background to support the association of circulating GDF15 at diagnosis with acute-phase reactants and BVAS and further with outcomes during the course of AAV.

We need to consider two aspects of the Cox analyses in the present study. One concerns variables that were significant in the multivariable Cox analyses for all-cause mortality and EKSK occurrence during follow-up, and the other concerns the relaxed statistical standards for variables in the univariable analyses included in the multivariate analyses. As for the predictors of mortality, the multivariable Cox analysis for all-cause mortality showed no independent predictor of death; however, BVAS and white blood cell count at diagnosis showed patterns fairly close to statistical significance (Table 2). This was not surprising because BVAS at diagnosis is known to be a major predictor of all-cause mortality [31,32], but the independent association pattern between white blood cell count at diagnosis and all-cause mortality was a slightly unfamiliar result. It could be assumed that white blood cell count at diagnosis reflects infectious diseases at diagnosis, which might be a critical risk factor for early mortality in patients with AAV [33]. However, in this study, patients with serious infections were excluded or the diagnosis was made by reapplying the classification criteria for AAV after the infection had completely disappeared; therefore, this assumption could not be established. Additionally, the multivariate Cox analysis for progression to ESKD suggested two predictors, serum creatinine level and ESR at diagnosis, similar to the results of previous studies [34,35]. Although circulating GDF15 ≥ 2208.5 pg/mL did not meet the strict cut-off of statistical significance criterion, its predictive potential can be evaluated as sufficiently high in that it can be compared to these prominent traditional risk factors.

As for variables with *p* < 0.1 adopted, in order not to miss key variables that did not reach statistical significance due to the small number of patients, in this study, the multivariable Cox analyses included variables with *p* < 0.1 in the univariable analyses. When blood urea nitrogen (*p* = 0.092) was excluded from the multivariate Cox analysis for progression to ESKD, both ESR and serum creatinine at diagnosis were still independently associated with progression to ESKD, and circulating GDF15 at diagnosis also exhibited a trend for its association. Therefore, we concluded that adopting *p* < 0.1 in the multivariable analysis did not have a decisive effect on the results.

Several confounding factors affect circulating GDF15 aside from pathological (inflammatory) stimuli. First, in terms of ageing, it was reported that circulating GDF15 levels were elevated in older individuals [36]. Similarly, in the present study, circulating GDF15 at diagnosis significantly correlated with age (r = 0.347, *p* = 0.002), as depicted in Figure 1. Additionally, patients with circulating GDF15 ≥ 3350.5 pg/mL (the cut-off for the highest tertile of BVAS at that time) were significantly older than those with circulating GDF15 <3350.5 pg/mL (74.5 vs. 60.0 years, *p* = 0.001). However, no significant correlation was found between age and BVAS at that time. When analysed using the multivariable linear regression analysis of age and BVAS for circulating GDF15, both age (standardized β, 0.328; *p* = 0.002) and BVAS (standardized β, 0.320; *p* = 0.002) were independently correlated with GDF15 without multicollinearity. Therefore, we can conclude that age has a communication link with circulating GDF15 but does not have a significant influence on the association between BVAS and GDF15.

Second, in terms of diabetes mellitus, it has been reported that circulating GDF15 levels are affected by type 2 diabetes mellitus and its systemic complications and metformin, one of its treatment medications [26,37]. To avoid the effects of diabetes and metformin, we excluded 17 patients with type 2 diabetes mellitus from the 79 patients and included only 62 patients without diabetes mellitus before analysing them again. Circulating GDF15 levels were also significantly correlated with BVAS (r = 0.380, *p* = 0.002). Similarly, the median BVAS and FFS values were 5.0, and the bottom of the highest tertile of the BVAS was calculated as 12. Upon ROC curve analysis, when the cut-off of circulating GDF15 for the highest tertile of BVAS was set at 3350.5 pg/mL, the sensitivity and specificity were 38.1% and 95.1%, respectively (AUC, 0.693; 95% CI: 0.550, 0.837). When patients were divided into two groups according to circulating GDF15 of ≥3350.5 pg/mL, the highest tertile of BVAS was identified more frequently in patients with circulating GDF15 ≥ 3350.5 pg/mL than those with circulating GDF15 <3350.5 pg/mL (75.0% vs. 20.0 *p* = 0.002). Additionally, patients with circulating GDF15 ≥ 3350.5 pg/mL exhibited a significantly higher risk for the highest tertile of BVAS than those with circulating GDF15 <3350.5 pg/mL (RR, 12.000; 95% CI: 2.255, 63.861) (Figure S1). Therefore, we conclude that the ability of circulating GDF15 at diagnosis to predict the highest tertile of BVAS remained significant, regardless of the presence of diabetes mellitus in patients with AAV.

Third, in terms of obesity, it was reported that GDF15 might be affected by body weight and associated with body mass index disproportionally [38,39]. In this study, circulating GDF15 was not significantly correlated with body mass index (r = −0.084, *p* = 0.462). A U-shaped curve was observed when drawing a two-dimensional scatter curve of circulating GDF15 and body mass index (Figure S2). Because obesity is defined as a body mass index ≥ 25.0 kg/m^2^ [40], we divided the patients into two groups according to a body mass index ≥ 25.0 kg/m^2^. Among the 15 patients with a body mass index ≥ 25.0 kg/m^2^, no significant correlation was found between circulating GDF15 and body mass index.

However, we demonstrated that among the 64 patients with a body mass index <25.0 kg/m^2^, circulating GDF15 was inversely correlated with body mass index (r = −0.248, *p* = 0.048), which was consistent with the results of a previous study [38]. Therefore, we concluded that circulating GDF15 could inversely reflect body mass index in non-obese patients; however, body mass index seemed to have no significant influence on the clinical significance of circulating GDF15 in this study.

On the other hand, when we evaluated the rate of all-cause mortality among three subtypes of AAV, we found that five patients with MPA and one patient with GPA died during the disease course of AAV but none with EGPA. As such, we excluded 16 patients with EGPA and included only 63 patients with MPA or GPA in the additional subgroup analyses regarding the mortality predictability of circulating GDF15. Among 63 patients with MPA or GPA, the highest tertile of BVAS was set as BVAS ≥ 11, and 24 belonged to the group of the highest tertile of BVAS at diagnosis. The ROC curve for the highest tertile of BVAS showed a significant AUC of 0.755 (CI: 0.631, 0.880). The optimal cut-off of circulating GDF15 was determined as 1075.0 pg/mL (sensitivity 75.0%, and specificity 66.7%), and patients with circulating GDF15 ≥ 1075.0 pg/mL exhibited a significantly higher risk for the highest tertile of BVAS than those with circulating GDF15 <1075.0 pg/mL (RR, 6.000; 95% CI: 1.921, 18.738). Additionally, upon ROC curve analysis for all-cause mortality, the optimal cut-off of circulating GDF15 was set as 2239.5 pg/mL (sensitivity 66.7% and specificity 73.7%), and we found significant differences in the rate of all-cause mortality during follow-up between patients with circulating GDF15 ≥ 2239.5 pg/mL and those without (RR, 6.500; 95% CI: 1.990, 21.234). Summarizing the results mentioned above, no significant differences in the results were observed between the analyses including only patients with MPA/GPA and those including all patients with MPA/GPA/EGPA. Similar results were obtained regarding the utility of circulating GDF15 in predicting ESKD progression in another analysis including patients with MPA/GPA and those with all subtypes of AAV. Therefore, particularly given patients who may be classified into two subtypes of AAV at the same time, those in whom the exact classification of one subtype of AAV may be difficult, and further those whose subtype of AAV changes during the disease course of AAV in real clinical settings, we believe that it would be reasonable to elucidate the usefulness of circulating GDF15 for patients with AAV regardless of its subtypes.

This study has merit in that this is the first to demonstrate that circulating GDF15 at diagnosis could estimate BVAS and potentially foresee all-cause mortality progression of ESKD during the disease course in patients with AAV.

This study has several limitations. Most importantly, there are two critical issues; one is that the number of patients was not sufficient to generalize the results of this study and apply them to patients newly diagnosed with AAV in real clinical practice, and the other is that this study was conducted retrospectively by selecting patients from an observational cohort of Korean patients with AAV and using their stored sera. Additionally, confounding factors affecting circulating GDF15, such as food intake, hyperglycaemia, informal use of metformin, and strength of stress in each individual, could not be controlled in this study. The lack of serial measurement of circulating GDF15 at different time points after diagnosis was another limitation. Nevertheless, these issues can be offset by the characteristics of the pilot study. We believe that a future prospective study including more patients with AAV and those with other autoimmune diseases through multicentre or multinational cooperation and serial measurements of circulating GDF15 will provide more reliable and dynamic information on its clinical usefulness in patients with AAV.

## 5. Conclusions

In the present study, we demonstrated that among patients with AAV, circulating GDF15 at diagnosis has clinical implications for estimating BVAS and the potential to predict all-cause mortality and progression to ESKD during the disease course in patients with AAV.

## Figures and Tables

**Table 1:** Characteristics of patients with AAV (N = 79).

Variables	Values
At the time of AAV diagnosis	
Demographic data	
Age (years)	64.0 (52.0–74.0)
Male sex (N, (%))	32 (40.5)
Female sex (N, (%))	47 (59.5)
Ex-smoker (N, (%))	3 (3.8)
Body mass index (kg/m^2^)	22.4 (20.8–24.7)
AAV subtype (N, (%))	
MPA	39 (49.4)
GPA	24 (30.4)
EGPA	16 (20.3)
ANCA positivity	
MPO-ANCA titre	0 (0–32.0) mean 27.2
PR3-ANCA titre	0 (0–0)
MPO-ANCA (or P-ANCA)-positive (N, (%))	45 (57.0)
PR3-ANCA (or C-ANCA)-positive (N, (%))	12 (15.2)
Both ANCA-positive (N, (%))	3 (3.8)
ANCA-negative (N, (%))	25 (31.6)
AAV-specific indices	
BVAS	5.0 (3.0–17.0)
FFS	0 (0–1.0)
Systemic items of BVAS (N, (%))	
General manifestation	17 (21.5)
Cutaneous manifestation	12 (15.2)
Mucous and ocular manifestation	7 (8.9)
Otorhinolaryngologic manifestation	41 (51.9)
Pulmonary manifestation	49 (62.0)
Cardiovascular manifestation	9 (11.4)
Gastrointestinal manifestation	0 (0)
Renal manifestation	38 (48.1)
Nervous systemic manifestation	26 (32.9)
Comorbidities (N, (%))	
Type 2 diabetes mellitus	17 (21.5)
Hypertension	26 (32.9)
Dyslipidaemia	14 (17.7)
Acute-phase reactants	
ESR (mm/hr)	21.0 (7.0–74.8)
CRP (mg/L)	3.6 (0.9–28.6)
Laboratory results	
White blood cell count (/mm^3^)	7610.0 (5960.0–10,560.0)
Haemoglobin (g/dL)	12.0 (10.2–13.6)
Platelet count (×1000/mm^3^)	243.5 (192.3–354.8)
Fasting glucose (mg/dL)	94.5 (87.8–109.3)
Blood urea nitrogen (mg/dL)	19.4 (13.9–28.7)
Serum creatinine (mg/dL)	0.8 (0.6–1.6)
Total serum protein (g/dL)	6.8 (6.3–7.3)
Serum albumin (g/dL)	4.2 (3.6–4.4)
Circulating GDF15 (pg/mL)	995.0 (549.0–2211.0)
During the disease course	
Poor outcome (N, (%)	
All-cause mortality	6 (7.6)
ESKD	20 (25.3)
Follow-up duration based on each poor outcome (months)	
All-cause mortality	26.7 (12.1–45.7)
ESKD	25.8 (9.0–41.2)
Medications	
Glucocorticoids	78 (98.7)
Cyclophosphamide	52 (65.8)
Rituximab	16 (20.3)
Mycophenolate mofetil	20 (25.3)
Azathioprine	48 (60.8)
Tacrolimus	7 (8.9)
Methotrexate	3 (3.8)

Values are expressed as a median (25~75 percentile) or N (%). ANCA: antineutrophil cytoplasmic antibody; AAV: ANCA-associated vasculitis; MPA: microscopic polyangiitis; GPA: granulomatosis with polyangiitis; MPO: myeloperoxidase; P: perinuclear; PR3: proteinase 3; C: cytoplasmic; BVAS: Birmingham Vasculitis Activity Score; FFS: the Five-Factor Score; ESR: erythrocyte sedimentation rate; CRP: C-reactive protein; ESKD: end-stage kidney disease.

**Table 2:** Cox hazards model analyses of variables at diagnosis for all-cause mortality or ESKD during follow-up in patients with AAV (including only the variables with statistical significance upon univariable analysis).

**All-Cause Mortality**									
**Variables**	**Univariable**			**Multivariable** **(Serum GDF15)**			**Multivariable** **(Serum GDF15 ≥ 2239.5 pg/mL)**		
	**HR**	**95% CI**	***p* Value**	**HR**	**95% CI**	***p* Value**	**HR**	**95% CI**	***p* Value**
Age	1.098	0.997, 1.208	0.056	1.125	0.894, 1.416	0.314	1.124	0.902, 1.402	0.299
BVAS	1.077	0.995, 1.167	0.066	0.819	0.651, 1.029	0.087	0.805	0.639, 1.015	0.060
CRP (mg/L)	1.016	1.000, 1.033	0.052	1.042	0.988, 1.100	0.130	1.043	0.989, 1.101	0.122
White blood cell count (/mm^3^)	1.130	1.021, 1.250	0.018	1.374	0.994, 1.899	0.054	1.490	1.000, 2.221	0.050
Haemoglobin (g/dL)	0.604	0.388, 0.941	0.026	1.206	0.367, 3.970	0.757	1.210	0.368, 3.978	0.753
Total serum protein (g/dL)	0.390	0.150, 1.016	0.054	0.815	0.111, 5.952	0.840	0.845	0.100, 7.154	0.877
Serum albumin (g/dL)	0.152	0.047, 0.490	0.002	0.110	0.005, 2.245	0.151	0.175	0.007, 4.694	0.299
Circulating GDF15 (pg/mL)	1.000	1.000, 1.001	0.016	1.000	0.999, 1.001	0.624			
Circulating GDF15 ≥ 2239.5 pg/mL	7.834	1.431, 42.890	0.018				4.994	0.106, 236.015	0.414
**End-Stage Kidney Disease**									
**Variables**	**Univariable**			**Multivariable** **(Serum GDF15)**			**Multivariable** **(Serum GDF15 ≥ 2208.5 pg/mL)**		
	**HR**	**95% CI**	***p* value**	**HR**	**95% CI**	***p* value**	**HR**	**95% CI**	***p* value**
Female sex	4.123	1.208, 14.072	0.024	2.703	0.523, 13.980	0.236	3.311	0.632, 17.334	0.156
ESR (mm/hr)	1.017	1.006, 1.027	0.002	1.030	1.009, 1.051	0.005	1.029	1.008, 1.051	0.008
CRP (mg/L)	1.015	1.005, 1.025	0.005	0.985	0.956, 1.014	0.297	0.988	0.959, 1.019	0.452
Haemoglobin (g/dL)	0.737	0.594, 0.915	0.006	1.095	0.686, 1.749	0.704	1.161	0.723, 1.865	0.536
Blood urea nitrogen (mg/dL)	1.019	0.997, 1.041	0.092	0.974	0.905, 1.047	0.470	0.966	0.899, 1.038	0.350
Serum creatinine (mg/dL)	1.326	1.086, 1.619	0.006	2.922	1.316, 6.488	0.008	2.818	1.240, 6.408	0.013
Serum albumin (g/dL)	0.501	0.263, 0.956	0.036	1.798	0.402, 8.046	0.443	2.267	0.456, 11.277	0.317
Circulating GDF15 (pg/mL)	1.000	1.000, 1.000	<0.001	1.000	1.000, 1.000	0.135			
Circulating GDF15 ≥ 2208.5 pg/mL	5.006	2.066, 12.128	<0.001				3.979	0.916, 17.285	0.065

AAV: ANCA-associated vasculitis; ANCA: antineutrophil cytoplasmic antibody; BVAS: Birmingham Vasculitis Activity Score; ESR: erythrocyte sedimentation rate; CRP: C-reactive protein.

## Data Availability

The data used to support the findings of this study are included within the article and the Supplementary Materials.

## Supplementary Materials

The following are available online at https://www.mdpi.com/article/10.3390/jcm14061876/s1, Figure S1: Cut-off and relative risk of circulating GDF15 for the highest tertile of BVAS in patients with AAV and DM; Figure S2: Correlation analyses; Table S1: Cox hazards model analyses of variables at diagnosis for all-cause mortality or ESKD during follow-up in patients with AAV.
