# Supplementary material for: Circulating GDF15 May Estimate Vasculitis Activity and Predict Poor Outcomes During the Disease Course of ANCA-Associated Vasculitis
Source: J Clin Med. 2025 Mar 11;14(6):1876. doi: 10.3390/jcm14061876 (PMC11942900; doi:10.3390/jcm14061876)
Supplement: Supplementary file 1 [file jcm-14-01876-s001.zip › SUPPLEMENTARY TABLES1(GDF15&AAV).pdf]

Supplementary Table S1. Cox hazards model analyses of variables at diagnosis for all-cause mortality or ESKD during follow-up in patients with AAV

**ALL-CAUSE MORTALITY**									
**Variables**	**Univariable**			**Multivariable (Serum GDF15)**			**Multivariable (Serum GDF15 ≥ 2239.5 pg/mL)**		
	**HR**	**95% CI**	**P value**	**HR**	**95% CI**	**P value**	**HR**	**95% CI**	**P value**
Age	1.098	0.997, 1.208	0.056	1.125	0.894, 1.416	0.314	1.124	0.902, 1.402	0.299
Male sex	2.933	0.537, 16.015	0.214						
Ex-smoker	0.046	0.000, 1512453.731	0.728						
Body mass index (kg/m^2)	0.900	0.679, 1.193	0.464						
Type 2 diabetes mellitus	1.891	0.346, 10.329	0.462						
Hypertension	1.126	0.206, 6.150	0.891						
Dyslipidaemia	2.505	0.458, 13.692	0.289						
MPO-ANCA (or P-ANCA) positivity	4.501	0.524, 38.644	0.170						
PR3-ANCA (or C-ANCA) positivity	0.038	0.000, 552.366	0.504						
BVAS	1.077	0.995, 1.167	0.066	0.819	0.651, 1.029	0.087	0.805	0.639, 1.015	0.060
FFS	2.147	0.830, 5.551	0.115						
ESR (mm/hr)	1.017	0.995, 1.039	0.123						
CRP (mg/L)	1.016	1.000, 1.033	0.052	1.042	0.988, 1.100	0.130	1.043	0.989, 1.101	0.122
White blood cell count (/mm^3)	1.130	1.021, 1.250	0.018	1.374	0.994, 1.899	0.054	1.490	1.000, 2.221	0.050
Haemoglobin (g/dL)	0.604	0.388, 0.941	0.026	1.206	0.367, 3.970	0.757	1.210	0.368, 3.978	0.753
Platelet count (x1,000/mm^3)	1.002	0.998, 1.006	0.369						
Fasting glucose (mg/dL)	1.007	0.997, 1.016	0.187						
Blood urea nitrogen (mg/dL)	1.023	0.986, 1.061	0.221						
Serum creatinine (mg/dL)	1.257	0.846, 1.868	0.257						
Total serum protein (g/dL)	0.390	0.150, 1.016	0.054	0.815	0.111, 5.952	0.840	0.845	0.100, 7.154	0.877
Serum albumin (g/dL)	0.152	0.047, 0.490	0.002	0.110	0.005, 2.245	0.151	0.175	0.007, 4.694	0.299
Circulating GDF15 (pg/mL)	1.000	1.000, 1.001	0.016	1.000	0.999, 1.001	0.624			
Circulating GDF15 ≥ 2239.5 pg/mL	7.834	1.431, 42.890	0.018				4.994	0.106, 236.015	0.414
**END-STAGE KIDNEY DISEASE**									
**Variables**	**Univariable**			**Multivariable (Serum GDF15)**			**Multivariable (Serum GDF15 ≥ 2208.5 pg/mL)**		
	**HR**	**95% CI**	**P value**	**HR**	**95% CI**	**P value**	**HR**	**95% CI**	**P value**
Age	1.023	0.990, 1.058	0.171						
Female sex	4.123	1.208, 14.072	0.024	2.703	0.523, 13.980	0.236	3.311	0.632, 17.334	0.156
Ex-smoker	0.046	0.000, 475.971	0.514						
Body mass index (kg/m^2)	0.989	0.859, 1.138	0.876						
Type 2 diabetes mellitus	0.648	0.190, 2.213	0.489						

Hypertension	1.881	0.779, 4.540	0.160						
Dyslipidaemia	0.543	0.126, 2.342	0.413						
MPO-ANCA (or P-ANCA) positivity	2.200	0.843, 5.744	0.107						
PR3-ANCA (or C-ANCA) positivity	0.591	0.137, 2.550	0.481						
BVAS	1.033	0.986, 1.083	0.166						
FFS	1.333	0.787, 2.258	0.285						
ESR (mm/hr)	1.017	1.006, 1.027	0.002	1.030	1.009, 1.051	0.005	1.029	1.008, 1.051	0.008
CRP (mg/L)	1.015	1.005, 1.025	0.005	0.985	0.956, 1.014	0.297	0.988	0.959, 1.019	0.452
White blood cell count (/mm^3)	1.015	0.927, 1.110	0.752						
Haemoglobin (g/dL)	0.737	0.594, 0.915	0.006	1.095	0.686, 1.749	0.704	1.161	0.723, 1.865	0.536
Platelet count (x1,000/mm^3)	1.001	0.998, 1.004	0.648						
Fasting glucose (mg/dL)	1.003	0.993, 1.013	0.573						
Blood urea nitrogen (mg/dL)	1.019	0.997, 1.041	0.092	0.974	0.905, 1.047	0.470	0.966	0.899, 1.038	0.350
Serum creatinine (mg/dL)	1.326	1.086, 1.619	0.006	2.922	1.316, 6.488	0.008	2.818	1.240, 6.408	0.013
Total serum protein (g/dL)	1.264	0.661, 2.418	0.479						
Serum albumin (g/dL)	0.501	0.263, 0.956	0.036	1.798	0.402, 8.046	0.443	2.267	0.456, 11.277	0.317
Circulating GDF15 (pg/mL)	1.000	1.000, 1.000	<0.001	1.000	1.000, 1.000	0.135			
Circulating GDF15 ≥ 2208.5 pg/mL	5.006	2.066, 12.128	<0.001				3.979	0.916, 17.285	0.065

ESKD: end-stage kidney disease; AAV: ANCA-associated vasculitis; ANCA: antineutrophil cytoplasmic antibody; HR: hazard ratio; CI: confidence interval; MPO: myeloperoxidase; P: perinuclear; PR3: proteinase 3; C: cytoplasmic; BVAS: Birmingham vasculitis activity score; FFS: five-factor score; ESR: erythrocyte sedimentation rate; CRP: C-reactive protein.
