# Supplementary figures and images for: Circulating GDF15 May Estimate Vasculitis Activity and Predict Poor Outcomes During the Disease Course of ANCA-Associated Vasculitis
Source: J Clin Med. 2025 Mar 11;14(6):1876. doi: 10.3390/jcm14061876 (PMC11942900; doi:10.3390/jcm14061876)

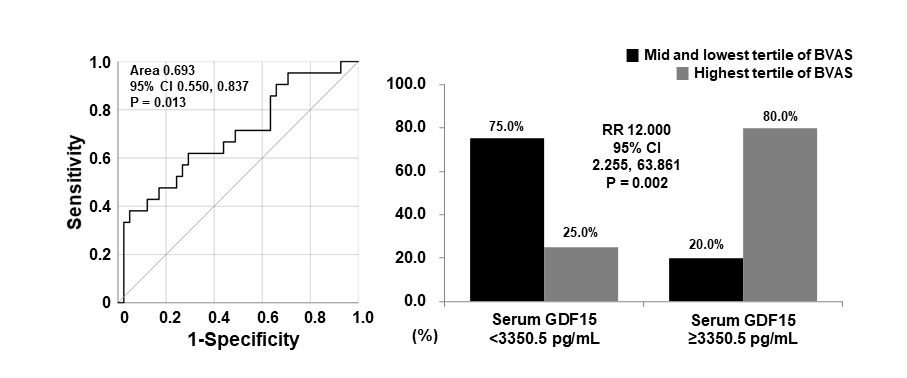

Supplement: Supplementary file 1 [file jcm-14-01876-s001.zip › SUPPLEMENTARY FIGURE1(GDF15&AAV).tif]

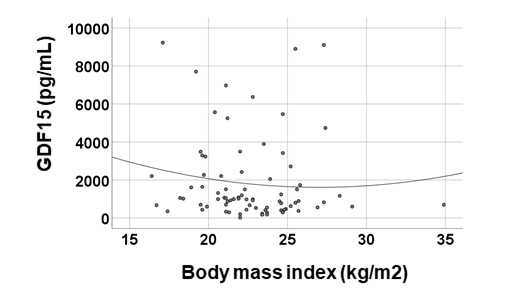

Supplement: Supplementary file 1 [file jcm-14-01876-s001.zip › SUPPLEMENTARY FIGURE2(GDF15&AAV).tif]
